# Supplementary material for: In Situ Vitrification of Lung Cancer Organoids on a Microwell Array
Source: Micromachines (Basel). 2021 May 28;12(6):624. doi: 10.3390/mi12060624 (PMC8227627; doi:10.3390/mi12060624)
Supplement: Supplementary file 1 [file micromachines-12-00624-s001.zip › micromachines-1139038-supplementary.pdf]

Table S1. Recipe of the lung cancer organoid culture media (LCOM)

Regents	Source	Catalog No.	Final concentration
DMEM/F12	ThermoFisher Scientific	11320-033	
GlutaMAX	Gibco	35050-061	1%(v/v)
HEPES	Gibco	15630-080	10 mM
Penicillin/Streptomycin	Hyclone	SV30010	1%(v/v)
B-27 Supplement	Invitrogen	17504044	2% (v/v)
N2 Supplement	Invitrogen	17502048	1% (v/v)
Nicotinamide	Selleckchem	1899	5 mM
N-Acetyl-L-cysteine	Selleckchem	S1632	1 mM
Y-27632	Selleckchem	S1049	10 μM
EGF	Peprotech	AF-100-15	50 ng/mL
SB202190	Selleckchem	S1077	3 μM
A83-01	Tocris	2939	5 μM
Forskolin	Selleckchem	2449	10 μM
Dexamethasone	Selleckchem	1322	3 nM

Table S2. Primer sequences for qPCR analysis of selected genes and control.

Primer name	Sequence（5’-3’）
GAPDH-F	GTCTGAACCATGAGAAGTATGA
GAPDH-R	CTTCCACGATACCAAAGTTGT
Bax-F	GTCAGCTGCCACTCGGAAA
Bax-R	AGTAACATGGAGCTGCAGAGGAT
Bcl-2-F	TCAGAGACAGCCAGGAGAAATCA
Bcl-2-R	CCTGTGGATGACTGAGTACCTGAA
Bcl-XL-F	ATGGCAGCAGTAAAGCAAGC
Bcl-XL-R	CGGAAGAGTTCATTCACTACCTGT
Bid-F	ACTGGTGTTTGGCTTCCTCC
Bid-R	ATTCTTCCCAAGCGGGAGTG
SOD1-F	CTGAAGGCCTGCATGGATTC
SOD1-R	CCAAGTCTCCAACATGCCTCTC
p53-F	CCCAAGCAATGGATGATTTGA
p53-R	GGCATTCTGGGAGCTTCATCT
